# Tone burst-evoked otoacoustic emissions in neonates: normative data

**DOI:** 10.1186/1472-6815-8-3

**Published:** 2008-04-17

**Authors:** Vicky Wei Zhang, Bradley McPherson, Zhi-Guo Zhang

**Affiliations:** 1 Centre for Communication Disorders, The University of Hong Kong, Hong Kong; 2 Department of Orthopaedics & Traumatology, and Department of Electrical & Electronic Engineering, The University of Hong Kong, Hong Kong

## Abstract

**Background:**

Tone-burst otoacoustic emissions (TBOAEs) have not been routinely studied in pediatric populations, although tone burst stimuli have greater frequency specificity compared with click sound stimuli. The present study aimed (1) to determine an appropriate stimulus level for neonatal TBOAE measurements when the stimulus center frequency was 1 kHz, (2) to explore the characteristics of 1 kHz TBOAEs in a neonatal population.

**Methods:**

A total of 395 normal neonates (745 ears) were recruited. The study consisted of two parts, reflecting the two study aims. Part I included 40 normal neonatal ears, and TBOAE measurement was performed at five stimulus levels in the range 60–80 dB peSPL, with 5 dB incremental steps. Part II investigated the characteristics of the 1 kHz TBOAE response in a large group of 705 neonatal ears, and provided clinical reference criteria based on these characteristics.

**Results:**

The study provided a series of reference parameters for 1 kHz TBOAE measurement in neonates. Based on the results, a suggested stimulus level and reference criteria for 1 kHz TBOAE measures with neonates were established. In addition, time-frequency analysis of the data gave new insight into the energy distribution of the neonatal TBOAE response.

**Conclusion:**

TBOAE measures may be a useful method for investigating cochlear function at specific frequency ranges in neonates. However, further studies of both TBOAE time-frequency analysis and measurements in newborns are needed.

## Background

Click evoked otoacoustic emissions (CEOAEs), as one type of transient evoked otoacoustic emission (TEOAE), have been widely used to assess the functioning of cochlear outer hair cells. Since the CEOAE click stimulus has a broad spectrum, and consequently can stimulate a broad frequency region of the cochlea in a single measurement, CEOAE measurement has been especially applied as a general tool in universal neonatal hearing screening (UNHS) programs. Another type of TEOAE – tone burst evoked OAEs (TBOAEs) – uses narrow bandwidth tone stimuli. This allows stimulus energy to be concentrated on a particular area of the basilar membrane and elicits a more frequency-specific cochlear response [1,2]. Fourier analysis of TBOAEs indicates that emission spectra are similar to that of the tone burst stimulus [3-5]. As to the research and clinical application of TBOAEs, studies have mainly focused on adult populations, and have been undertaken by few authors.

Compared with CEOAEs, TBOAEs at similar stimulus levels can achieve a stronger response level with a greater signal to noise ratio (SNR) in normal adult ears [6-8]. Also, the short- and long-term test-retest reliabilities for TBOAE were found to be acceptable when using high (76 dB peSPL) and mid (67 dB peSPL) stimulus levels [6]. Concerning the prevalence of TBOAEs, different studies have reported different findings. Liu et al. [9] noted that the prevalence rate for a 1 kHz TBOAE was 100% in 35 normal hearing adults. Similarly, Chan and McPherson [6] found a 1 kHz tone stimulus with high stimulus level could elicit TBOAEs in all normal hearing adults tested (30 ears). However, Probst et al. [4] reported that not all 28 tested adult ears responded to all tone burst stimuli (ranging from 0.5 to 3 kHz). They found the percentages of detected emissions for stimuli at 0.5, 1, 1.5 and 3 kHz were 36%, 82%, 100% and 93%, respectively.

As there is no standard protocol for TBOAE measurements, results presented in the literature are generally not directly comparable. A variety of different recording instruments, stimulus levels, stimulus center frequencies, stimulus rates, number of averages, and analysis windowing parameters have been employed. Table 1 summarizes studies and parameters that have been used for TBOAE measurement. According to studies of adult TBOAEs, high level stimuli are recommended as they save recording time [10], elicit a stronger response, and give higher wave reproducibility and reliability results [6] than lower level stimuli.

**Table 1:** Different stimulus parameters used in literature for TBOAE measurement

Authors	Equipment	Stimuli frequency range (kHz)	Sound levels	Stimulus repetition rate	Number of sweeps	Analysis window (ms)
Hauser et al., 1991 [29]	ILO88	0.5, 1, 2, 3, 4,5,6	10–40 dB nHL above mean hearing threshold	50	/	2.5–20
Xu et al., 1994 [43]	ILO88 Version 3.92	1, 2, 3	75, 59 and 37 dB SPL	50	260	5.5–20.5
Liu et al., 1996 [9]	ILO 92	0.5, 1, 2, 4	64–72 dB SPL	/	260	20
Prieve et al., 1996 [7]	Custom-based equipment	0.5, 1, 2, 4	20–80 ppe SPL	40	1024	30
Chan & McPherson, 2000 [6]	ILO 88; Version 5	0.5, 1, 1.5, 2, 3	≈55, 65 and 75 dB peSPL	50	260	1 kHz = 4.5–20 ms; 2 kHz = 2.5–20 ms; 3 kHz = 1.5–20 ms.
Moulin, 2000 [44]	ILO 88; Version 5.5	0.75 – 6	65 – 68 dB pe SPL	40 (for the 750 & 880 Hz stimuli); 60 (for greater frequencies)	260	25 (for the 750 & 880 Hz stimuli); 15 (for greater frequencies)
Epstein & Florentine, 2005 [45]	Custom-based equipment	/	10–70 dB SPL	12.2	/	20.48 ms
McPherson et al. 2006 [11]	ILO 288 version 5.6	1, 2, 3	75 dB pe SPL	50	60 or 120	1 kHz = 4.5–20 ms; 2 kHz = 2.5–20 ms; 3 kHz = 1.5–20 ms.

As to the use of TBOAEs for assessment in neonates and young children, few studies have been carried out [11,12]. It has been suggested that using lower frequency TBOAEs may better elicit a more robust OAE response than CEOAEs in the lower frequency region, and thus assist in reducing the often high referral rate found in traditional CEOAE neonatal hearing screening programs. Considering the potential role of TBOAEs for diagnostic cochlear assessment as well as in hearing screening for neonates, the present study examined neonatal TBOAE findings for a tone burst stimulus with a 1 kHz center frequency. The study consisted of two parts:

Part I: To determine the appropriate stimulus level for 1 kHz TBOAE response measurement, based on TBOAE prevalence rate and other considerations.

Part II: To investigate the characteristics of the 1 kHz TBOAE response in a large group of neonates and develop a set of reference criteria for 1 kHz TBOAE measurements.

## Methods

### Participants

A total of 395 neonates from a well-baby nursery were recruited at the Hong Kong Adventist Hospital on a voluntary basis. Written informed parental/legal guardian consent was obtained prior to subject enrolment in the research project. The mean test age was 2.54 days (SD = 1.0). 52.34% of subjects were male and 47.66% were female. All the subjects met study inclusion criteria as follows: older than 48 hours and younger than 7 days; gestation between 37 and 42 weeks; birth weight between 2.4 and 4.5 kg; normal birth history and first 24 hours after delivery; without apparent congenital defects; no history of high risk factors (family history of hearing loss; congenital or perinatal infection; anatomical malformation of head or neck; hyperbilirubinaemia; bacterial meningitis; severe perinatal asphyxia; convulsions; prolonged aminoglycoside usage and intracranial haemorrhage). Among the subject group, 745 CEOAE- and TBOAE-tested ears passed CEOAE hearing screening, and were included in the present studies. These ears were divided into two groups, and completed either Part I or Part II of the study. The Part I group consisted of 40 ears, and TBOAE measurement was performed at five stimulus levels in the range 60–80 dB peSPL. The Part II group comprised 705 ears, and the target stimulus level was fixed at 75 dB peSPL using the equipment software setting.

### Procedures

Any debris noted in the ear canal was removed using a cotton swab before inserting the OAE probe tip. The probe tip was checked for adequate fit and was refitted or changed as appropriate, if this was necessary. Newborns were tested while in natural sleep or a quiet state. The OAE test ear order for both CEOAE and TBOAE measures was random. All subjects initially had both CEOAE and TBOAE measures performed.

### Apparatus and parameters

All the measures were recorded in a non-sound treated room adjacent to the nursery ward at Hong Kong Adventist Hospital. The average ambient room noise level with OAE equipment in operation was under 45 dBA. OAE data were collected using the Echoport ILO 292 USB system with V6 software (Otodynamics Ltd., UK) installed in a laptop computer. A standard ILO system clinical neonatal probe was used and calibrated before every test session. In ILO software, the recorded OAE average responses were stored into two memory buffers, "A" and "B", for subsequent calculations.

Both CEOAEs and TBOAEs are recorded based on the nonlinear response mode. In order to compare findings with other reports, CEOAEs were recorded using the ILO default "QuickScreen" mode [13] for all study newborns. In this study, a passing CEOAE measurement was one that fulfilled the following CEOAE criteria: stimulus stability ≥ 75%; whole reproducibility ≥ 70%; at least three of five test frequency bands centered at 1, 1.5, 2, 3 and 4 kHz with signal to noise ratio (SNR) ≥ 3 dB. Similar criteria have been used by other researchers [11,14,15]. The recorded stimulus level of 75–80 dB equivalent sound pressure level (peSPL) in the ear canal was considered acceptable for CEOAE measurement.

Using the default ILO V6 software settings, TBOAEs were elicited with a two-cycle tone burst at 1 kHz with a raised cosine window and the stimulus length was fixed at 2 ms with plateau length defined by the stimulus duration. The analysis window and stimulus repetition rate for 1 kHz TBOAE measurements were 20.48 ms and approximately 50 Hz, respectively. In the Part I study, TBOAE measurement was performed at stimulus levels in the range 60–80 dB peSPL, with progressive 5 dB increment steps. Based on the results of the Part I study, the target stimulus level was set to 75 dB peSPL to elicit a TBOAE response in the subsequent Part II tests. An actual tone stimulus of 75–80 dB peSPL recorded in the ear canal was considered acceptable. A SNR = 3 dB was used to define a clear response at each frequency band [1]. Response stopping criteria for TBOAE measurements required at least 70 OAE stimuli presentations, or, if no clear TBOAE response at 70 presentations, up to 260 responses were obtained. The noise rejection level was set lower than 8 mPa (52 dB SPL). The data were analyzed using half-octave bands in ILO V6 software.

### Analysis

Analyses of Part I were used to determine the appropriate stimulus level for 1 kHz TBOAE measurement. This initial study used five stimuli levels (60, 65, 70, 75, 80 dB peSPL), and investigated the relationship between individual stimulus level and TBOAE parameters, including stimulus stability (which reflects changes in stimulus intensity occurring during the test and which should be greater than 75% of the initial stimulus amplitude [16]), whole reproducibility (which is calculated by the cross-correlation between A and B waveforms and which is used clinically as a quality index of the recorded OAE [16]), whole response amplitude and noise level, and the mean response level, SNR, and TBOAE response prevalence rate for 1, 1.5 and 2 kHz frequency components. Prevalence rate was defined using a criterion of SNR ≥3 dB [1]. A one way ANOVA test was used to determine whether there was any difference in TBOAE performance among the five stimulus levels.

The characteristics of 1 kHz TBOAEs from neonates with a target stimulus level of 75 dB peSPL were examined in Part II. Statistical analyses were performed using SPSS for Windows version 12.0 software. The level of statistical significance was set at 0.05, and was specified for each test as P.

Recorded TBOAEs were also analyzed in the time-frequency domain to explore the maximum energy range of the evoked 1 kHz TBOAE component, using both latency and frequency information. Wavelet transform (WT) was used as the time-frequency analysis technique because auditory processing in the cochlea is similar to a wavelet transform along the basilar membrane [17,18]. Comprehensive descriptions of WT for OAE recordings have been given by several researchers [19-24]. The continuous wavelet transform (CWT) of OAE level *x*(*n*) is calculated by $P_{WT}(n,\omega )=\int_{\tau }x(\tau )\cdot \sqrt{\omega /\omega_{0}}\cdot \psi^{*}(\omega /\omega_{0}.(\tau -n))d\tau$, where *n *and *ω *are, respectively, the time and frequency index, and *ψ*(·) is the mother wavelet function with central frequency *ω*_0_. The mother wavelet function *ψ*(·) used in this study was proposed by Tognola et al., which is a modulated cosine function *ϕ*(*t*) = (1/(1+ *t*^*β*^)) · cos(*α *· *t*) [23,24]. The time and frequency analysis of CEOAE signals have been shown to achieve the best results when *β *= 4 and *α *= 20 are used [23,24]. The squared magnitude of *P*_*WT*_(*n*, *ω*) is called the scalogram and it was used to give a time-frequency representation for OAE response level. Since the presence of OAE in the ILO system is determined by identification of emissions in two different buffers, A and B, the *x*(*n*) obtained was the average of the OAE levels from these two buffers. Wavelet analyses were conducted using MATLAB 7.0 software.

## Results

**Figure 1:**
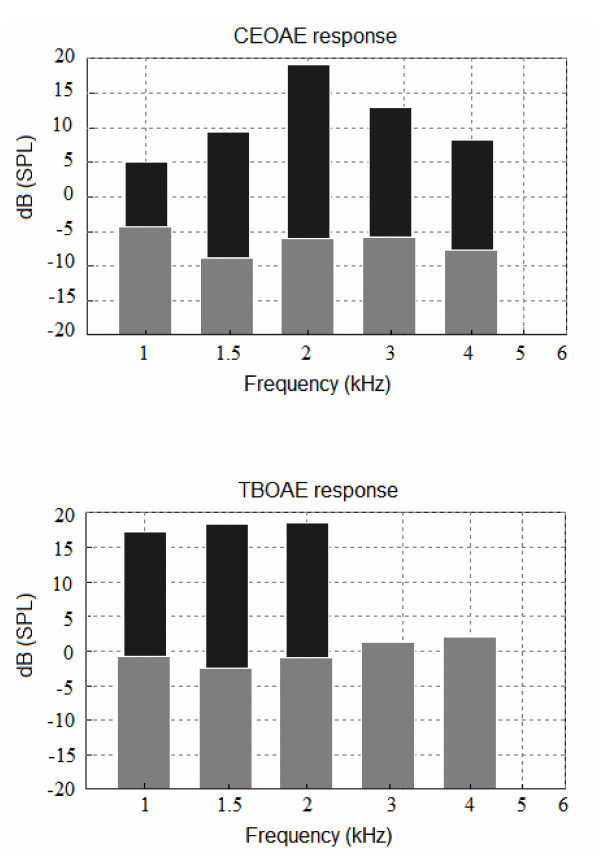
**Spectra of CEOAE and 1 kHz TBOAE recorded by ILO V6 software from the same neonatal ear**. The target stimulus level was 75 dB peSPL, and the recorded mean stimulus level in the ear canal was 76.9 dB peSPL. Black depicts the evoked OAE response, and grey depicts the noise floor.

In total 745 ears from neonates without any risk factor for hearing disorder and who had passed CEOAE screening test were included in this analysis. Response levels were averaged in dB SPL. A 1 kHz tone burst stimulus with a target level of 75 dB peSPL usually could evoke a TBOAE response in the 1 to 2 kHz frequency range, using a half-octave analysis (figure 1). Therefore, the following summary of mean emission response, SNR and prevalence rate findings will include results in the 1, 1.5 and 2 kHz half-octave bands.

### Part I: Relationship between stimulus level and 1 kHz TBOAE performance

**Figure 2:**
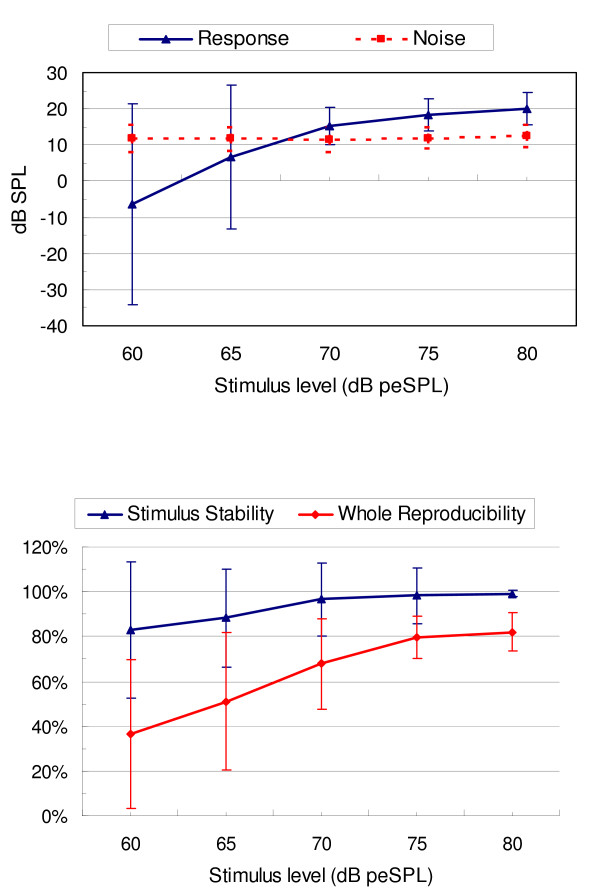
**Relationship between stimulus levels and 1 kHz TBOAE performance (n = 40 ears)**. The top figure illustrates mean (± SD) response level and noise level as a function of 1 kHz tone burst stimulus levels. The bottom figure shows the relationship between stimulus levels and mean (± SD) for stimulus stability and whole reproducibility TBOAE parameters.

The mean response levels and noise levels as functions of tone burst stimulus levels in 40 ears are illustrated in figure 2 (the top figure). The TBOAE response levels increased with increasing stimulus levels, but the noise levels remained similar. At 60 or 65 dB peSPL stimulus levels, the mean response was lower than the noise floor, and hence the corresponding mean SNR was less than 3 dB. However, 1 kHz TBOAE stimulus levels at 70, 75 and 80 dB peSPL could evoke a clear overall mean response, at 15.35 dB, 18.46 dB, and 20.11 dB, respectively. A one way ANOVA test showed that there was no significant difference for overall mean response for the 1 kHz TBOAE between any two of these three stimulus levels (P > 0.05).

Figure 2 (the bottom figure) analyzed the whole reproducibility and stimulus stability for 1 kHz TBOAE measurement at different stimulus levels. Mean reproducibility was greater than 60% only when the stimulus level reached 70 dB peSPL or above. Significant differences for reproducibility were found between the lower stimulus levels (60 or 65 dB peSPL) and higher levels (70, 75 or 80 dB peSPL) (P < 0.05). In addition, the analysis showed that mean stimulus stability was greater than 80% for all the five stimulus levels, but a larger variation in stability was found at 60 or 65 dB peSPL.

For 1, 1.5 and 2 kHz frequency components, the relationship between the SNR of a 1 kHz TBOAE and stimulus levels is illustrated in table 2. The mean SNR was greater than 3 dB at 1 kHz frequency band when the stimulus level was higher than 70 dB peSPL. All stimulus levels could provide a SNR ≥ 3 dB at 1.5 kHz, but the standard deviations found with 60 and 65 dB peSPL were also higher than other stimulus levels for this frequency range. At 2 kHz, a stimulus level of 70 dB peSPL or lower could not provide mean SNR ≥ 3 dB.

**Table 2:** Relationship between stimulus levels and SNR for 1 kHz TBOAE at 1, 1.5 and 2 kHz frequency components (n = 40 ears)

Stimulus level (dB peSPL)	1 kHz TBOAE SNR (dB)					
	
	1 kHz		1.5 kHz		2 kHz	
	
	Mean	SD	Mean	SD	Mean	SD
60	-.95	8.22	6.08	8.45	-4.34	5.68
65	2.07	7.48	9.62	7.33	-1.09	5.43
70	5.28	5.42	13.38	5.49	2.43	6.63
75	7.74	4.15	15.63	4.61	6.99	5.74
80	8.20	4.38	16.58	5.50	9.32	6.31

**Figure 3:**
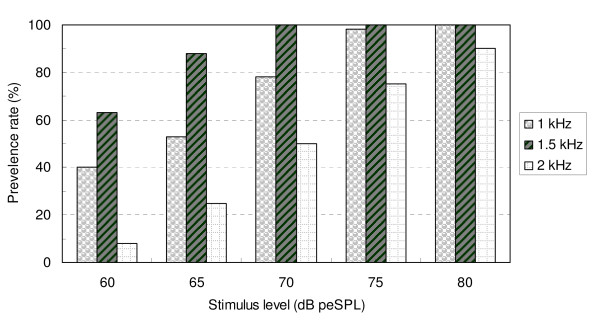
Prevalence of a response (percentages) at each stimulus level and for each frequency component for 1 kHz TBOAE stimulus (n = 40 ears).

The percentages for TBOAE prevalence at each stimulus level and for each frequency component are illustrated in figure 3. Decreasing the stimulus level to 60 or 65 dB peSPL resulted in an overall reduction in the prevalence rate for all three frequency bands. If an acceptable prevalence for 1 kHz TBOAE is considered to be 90% or above for 1 and 1.5 kHz frequency components then 75 or 80 dB peSPL is the best choice for target stimulus level.

### Part II: Characteristics of 1 kHz TBOAEs

Totally, 705 ears were recruited in this second part of the research program. Based on the results of Part I, the target stimulus level for the 1 kHz TBOAE was set to 75 dB peSPL, and a recorded stimulus level of 75–80 dB peSPL in the ear canal was considered acceptable. The mean recorded stimulus level for the TBOAE measurements was 77.1 dB peSPL (SD = 1.7).

The overall mean response level and the overall mean reproducibility for the recorded 1 kHz TBOAEs were 18.43 dB (SD = 7.73) and 74.54% (SD = 15.8), respectively. The mean stimulus stability and mean test duration per ear for 1 kHz TBOAE measurement was 96.82% (SD = 8.84) and 57.2s (SD = 37.3), respectively. There was no statistically significant difference concerning ear of test (P > 0.05) or gender effect (P > 0.05) on overall mean response level for TBOAEs. In addition, a one way ANOVA test did not find these three parameters were affected by the test age (from 2 to 7 days) of the neonates.

**Figure 4:**
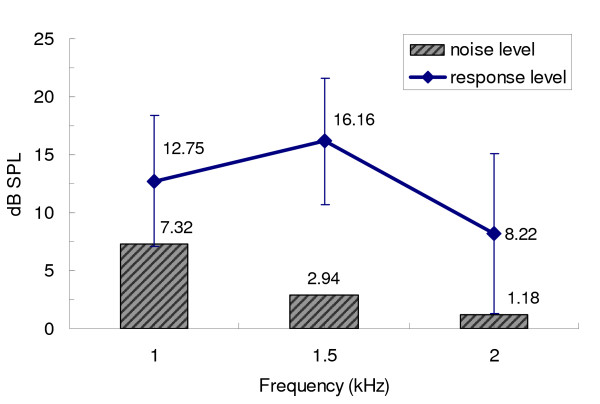
Mean (± SD) TBOAE response and noise level at different frequency bands (n = 705 ears).

The mean response and noise for TBOAEs at different frequency bands are illustrated in figure 4. A one way ANOVA test showed that mean noise level at 1 kHz was significantly higher than in the other two frequency bands (P < 0.05). The mean TBOAE response at 1 kHz was significantly higher than that of 2 kHz (P < 0.05), but lower than 1.5 kHz (P < 0.05). The mean SNRs for TBOAE at 1, 1.5, and 2 kHz frequency bands were 5.4 dB (SD = 5.5), 13.2 dB (SD = 5.4) and 7.0 dB (SD = 6.4), respectively. The SNR differences among frequency bands were significant. The "confidence (%)" reported on the V6 screen is equivalent to the band-limited "reproducibility" in previous software, which was calculated from the correlation between waveforms. Since this variable is a function of the SNR in a given frequency band, this study will only analyze the SNRs, instead of band-limited "confidence (%)."

In present study, SNR ≥ 3 dB was used as a criterion defining a detectable TBOAE response at each frequency band. The highest prevalence rate for 1 kHz TBOAE measurement occurred at 1.5 kHz, with a prevalence rate of 97.4% (687 ears). In contrast, the 1 kHz tone burst elicited a response in 556 ears (78.9%) at 1 kHz. The prevalence rate for TBOAEs at 2 kHz was 77.6% (547 ears). A chi-square test indicated that differences in TBOAE prevalence rate were significant between the 1 and 1.5 kHz frequency ranges (*χ*^2^(1) = 116.57, p < 0.05), as well as 1.5 and 2 kHz bands (*χ*^2^(1) = 127.25, p < 0.05). There was no significant difference between 1 and 2 kHz frequency bands (*χ*^2^(1) = 0.27, p > 0.05).

**Figure 5:**
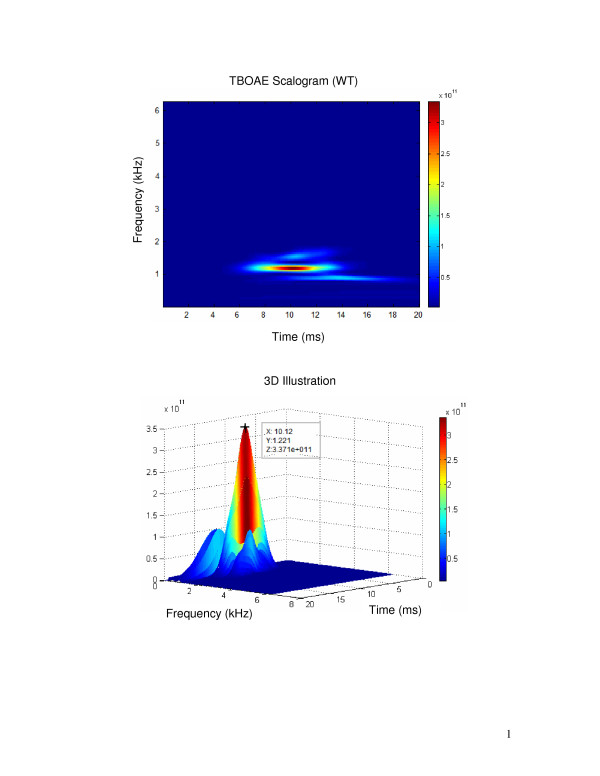
**Time-frequency distribution of a 1 kHz TBOAEs response from a neonatal ear using WT method**. Colour bar on the right side indicates the range of intensity values of TBOAE energy. TBOAE scalogram indicates that the highest intensity range locations are at approximately 1 kHz and 10 ms (top of the figure). 3D illustration shows the peak value of 1 kHz TBOAE energy, occurring at 10.12 ms and 1221 Hz (bottom of the figure).

Figure 5 illustrates the time-frequency distribution of a 1 kHz TBOAE response from a neonatal ear using the WT method. The TBOAE scalogram shows the highest intensity range is located around 1 kHz and 10 ms post-stimulus (the top figure). In order to obtain a more accurate peak value, the bottom figure plots a 3D illustration that shows the peak of 1 kHz TBOAE energy occurring at 10.12 ms and 1221 Hz. Among all the 705 tested ears, 458 data sets showed a clear 1 kHz TBOAE response with SNR ≥ 3 dB for the 1, 1.5 and 2 kHz frequency bands. Based on these 458 data sets, the mean time for occurrence of 1 kHz TBOAE maximum energy was 10.17 ms (SD = 1.61) and at 1325.3 Hz (SD = 222.46).

## Discussion

**Figure 6:**
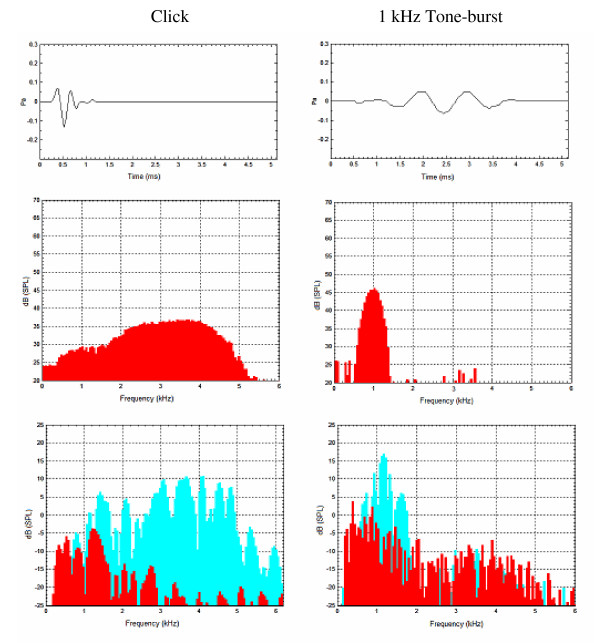
**Click and 1 kHz tone burst stimuli, as well as corresponding CEOAE and 1 kHz TBOAE responses**. The top figure shows the stimulus waveforms of click and 1 kHz tone burst sounds. The middle figure shows the stimulus spectrums of click and 1 kHz tone burst sounds. The bottom figure illustrates the response spectrums of CEOAE and 1 kHz TBOAE recordings. Blue depicts the evoked OAE response, and red depicts the noise floor. The figure shows that a tone burst stimulus has a narrow bandwidth compared with a click sound. Fourier analysis of TBOAEs indicates that emission spectra are similar to those of the tone burst stimulus, but the evoked energy of a 1 kHz TBOAE response may have a spectral spread and is not confined to the 1 kHz frequency range.

It has been reported that the 500 to 1500 Hz components of a CEOAE response are likely to be contaminated by low frequency noise in a normal hearing individual [25-28]. The mean prevalence rate for CEOAEs in the 1 kHz frequency range is below 40% in neonates. [12]. Other reasons that the 1 kHz frequency component of CEOAE is difficult to evoke may be due to the acoustic characteristics of the click sound and the shortened time window of the "QuickScreen" mode routinely used in the CEOAE neonatal hearing screening test. Compared with click sound stimuli, a tone burst stimulus has greater frequency specificity because of its acoustic characteristics [16] (figure 6). Therefore, a 1 kHz tone burst may be superior to a click stimulus in the low frequency test range, improving the SNR and increasing likelihood of OAE detection. As a potential tool for the assessment of cochlear activity, however, details of TBOAE performance have not been reported, nor have TBOAEs been routinely used, for pediatric populations. The present study sought to determine reference criteria for TBOAE measurement when the stimulus center frequency was 1 kHz and to explore the characteristics of 1 kHz TBOAEs obtained from neonates.

From the Part I study, recommendations regarding the setting of appropriate TBOAE stimulus levels for newborns were made, based on TBOAE performance – which included overall mean response, whole reproducibility, stimulus stability, as well as the prevalence rate and SNR in different frequency ranges. With decreased stimulus level, all of these parameters had a trend towards reduced values. Figure 2 showed that TBOAEs evoked by lower stimulus levels (60 and 65 dB peSPL) resulted in lower whole reproducibility (< 60%), lower stimulus stability (< 90%), and unacceptable lower overall mean response (SNR < 0 dB). Stimuli levels of 70, 75 and 80 dB peSPL did not show significant differences among each other on the above three parameters. However, setting stimulus levels at 70 dB peSPL or below made for a large variation in some TBOAE parameters, such as reproducibility and stimulus stability. Also, it was demonstrated that when the stimulus level was 75 dB peSPL or above the prevalence rate for detectable 1 kHz TBOAEs was higher than 90% in the 1 and 1.5 kHz frequency bands. Therefore, based on all the above results, in the Part II study the target tone burst stimulus level was set at 75 dB peSPL using the ILO system – software. The real stimulus level recorded in situ – which was measured using the ILO system ranged from 75 to 80 dB peSPL and this was viewed as an acceptable stimulus range.

The Part II study explored several parameters of 1 kHz TBOAEs recorded from 705 neonatal ears using the predetermined 75 dB peSPL stimulus level. The overall mean response level, whole reproducibility, and stimulus stability for 1 kHz TBOAEs were 18.43 dB (SD = 7.73), 74.5% (SD = 15.8), and 96.8% (SD = 8.8), respectively. All the above parameters were in the range of a previous, smaller study [12]. A one way ANOVA test did not find test age of the neonate had an effect on these three parameters or on the mean response and SNR at each frequency band. The overall mean response level for TBOAEs in the present study was higher than noted in findings for an adult population [6,7,29].

The mean SNRs for 1 kHz TBOAE at the three analyzed frequency bands were all greater than 3 dB, but the SNR at 1 kHz was significantly lower than that at 1.5 and 2 kHz frequency bands, even though the mean TBOAE response at 1 kHz was significantly greater than that at 2 kHz. It was also found that although the stimulus frequency was centered at 1 kHz, the prevalence rate in the 1 kHz frequency band was lower than that at 1.5 kHz. The main reason for these findings relates to greater noise in the 1 kHz frequency band (figure 4), which may overlay a substantial but weaker TBOAE response. In addition, a default low frequency filter setting in the ILO system and "QuickScreen" mode with a shortened response window will reduce the noise levels at lower frequencies, but also reduce the OAE response as well [7,30]. An additional reason that SNR and prevalence rate of TBOAEs in the 1 kHz frequency band were lower than those at 1.5 kHz may be that the 1 kHz tone burst stimulus may not evoke a response solely confined to the 1 kHz frequency range in the cochlear partition (figure 6). There may also be some influence across TBOAE channels, which may not function as independently as expected [7,31,32]. This possibility was supported by our finding that the mean of the maximum evoked energy of the neonatal 1 kHz TBOAE response was located at around 1.3 kHz. A final reason for the finding of a greater response at 1.5 kHz may be related to the ILO results display manner. Since the half octave display sums the number of responses across a frequency region ¼ octave above and below the centre frequencies, the lower and upper frequencies for the target 1 kHz frequency half octave band would be around 850 and 1200 Hz. Therefore, based on a half-octave analysis, the evoked highest energy (with a mean of 1325 Hz) may have been included in the 1.5 kHz analysis half octave band. For the 2 kHz frequency component, the prevalence rate of TBOAEs was 77.6%, which was lower than that of CEOAEs with 99.3%. Therefore, a 1 kHz tone burst stimulus might be more suitable than a click stimulus in providing restricted low frequency information (lower than 2 kHz) for a pediatric population, and the 1 kHz TBOAE pass criteria may need to focus on both the 1 and 1.5 kHz frequency bands.

Based on all the above results, suggested criteria for a valid 1 kHz TBOAE measurement for newborns are: stimulus level range 75–80 dB peSPL; stimulus stability ≥ 75%; whole reproducibility ≥ 60%; SNR ≥ 3 dB at 1 and/or 1.5 kHz. By using these criteria as a reference, supplementary information in the lower frequency region may be provided by 1 kHz TBOAE measurement. When combined 1 kHz TBOAE plus CEOAE measures are recorded using these criteria, initial studies suggest the pass rate for neonatal hearing screening may be significantly improved compared with conventional CEOAE-only screening [33].

The results of time-frequency analysis using the WT method provided further insight into the TBOAE energy range in both time and frequency domains. Time-frequency analysis of CEOAE has been undertaken by a number of researchers [21-23,34-37]. Several studies have demonstrated longer latencies for lower frequencies [13,37-39]. However, there were no studies noted concerning the time-frequency analysis and related range of maximum energy for TBOAE responses. The present study gives the first report on time-frequency analysis for TBOAEs evoked by a 1 kHz tone burst in a neonatal population. With a recorded mean stimulus level of 77 dB peSPL, the mean latency of the maximum energy of 1 kHz TBOAEs was 10.17 ms (SD = 1.61), and the mean of peak energy occurred at 1325.3 Hz (SD = 222.46). The energy distribution of the 1 kHz TBOAE provides a potential reference parameter for newborns, and it is valuable when compared with other reports in adults. Prieve et al. [7] showed the mean delay for a 1 kHz tone burst at 70 dB ppe SPL was approximately 13 ms. Similarly, Wit and Ritsma [39] noted that the mean latency for a 1 kHz tone burst presented at 50 dB SPL was 11 ms. These results were also within the range of latencies reported by others [1,40-42], although the TBOAE stimulus levels were not specified in some studies. Bray [13] reported that the post stimulus response time for a 1 kHz TBOAE was about 10 ms in adults, and this result was similar to our current findings in neonates. Further work may study different TBOAE stimulus frequencies and compare the results with the corresponding frequency components of CEOAE measurement. Time-frequency distributions may in future be used in distinguishing normal and pathological ears by analyzing the energy range of the CEOAE/TBOAE response using both latency and frequency parameters. With the development of new techniques, automatic evaluation of TBOAE responses by time-frequency distribution should be possible and may enhance the accuracy of the information provided by OAE equipment.

## Conclusion

The present study provided a reference for 1 kHz TBOAE stimulus parameters and possible pass/refer criteria for neonatal screening. The characteristics of 1 kHz TBOAE measurement parameters were outlined, as a possible contribution to the clinical assessment of TBOAEs in neonates and infants. TBOAE time-frequency analysis gave a new insight into the energy distribution of the response, and the findings may lead to the development of new OAE pass/fail criteria. Further studies of both time-frequency analysis and TBOAE measurements are needed to compare results between normal and pathological ears in newborns. TBOAE measures may be useful in investigating cochlear function at specific frequency ranges, identifying a change in hearing status and in monitoring deteriorating audition in infants.

## Competing interests

The author(s) declare that they have no competing interests.

## Authors' contributions

VWZ designed the study, carried out the literature research, data collection, conducted data analysis, manuscript preparation and manuscript review. BM conceived and outlined the overall research direction, contributed to design and project coordination, carried out the manuscript review. ZGZ assisted in the biomedical engineering aspects of the project and carried out the manuscript review. All authors read and approved the final manuscript.

## Pre-publication history

The pre-publication history for this paper can be accessed here:


